# The Zn(II)2Cys6-Type Transcription Factor ADA-6 Regulates Conidiation, Sexual Development, and Oxidative Stress Response in *Neurospora crassa*

**DOI:** 10.3389/fmicb.2019.00750

**Published:** 2019-04-10

**Authors:** Xianyun Sun, Fei Wang, Nan Lan, Bo Liu, Chengcheng Hu, Wei Xue, Zhenying Zhang, Shaojie Li

**Affiliations:** ^1^State Key Laboratory of Mycology, Institute of Microbiology, Chinese Academy of Sciences, Beijing, China; ^2^College of Life Sciences, University of Chinese Academy of Sciences, Beijing, China; ^3^College of Food Science and Engineering, Qilu University of Technology, Jinan, China

**Keywords:** conidiation, *ada-6*, sexual development, oxidative stress response, *Neurospora crassa*

## Abstract

Conidiation and sexual development are critical for reproduction, dispersal and better-adapted survival in many filamentous fungi. The *Neurospora crassa* gene *ada-6* encodes a Zn(II)2Cys6-type transcription factor, whose deletion resulted in reduced conidial production and female sterility. In this study, we confirmed the positive contribution of *ada-6* to conidiation and sexual development by detailed phenotypic characterization of its deletion mutant and the complemented mutant. To understand the regulatory mechanisms of ADA-6 in conidiation and sexual development, transcriptomic profiles generated by RNA-seq from the Δ*ada-6* mutant and wild type during conidiation and sexual development were compared. During conidial development, differential expressed genes (DEGs) between the Δ*ada-6* mutant and wild type are mainly involved in oxidation-reduction process and single-organism metabolic process. Several conidiation related genes are positively regulated by ADA-6, including genes that positively regulate conidiation (*fluffy* and *acon-3*), and genes preferentially expressed during conidial development (*eas*, *con-6*, *con-8*, *con-10*, *con-13*, *pcp-1*, and NCU9357), as the expression of these genes were lower in the Δ*ada-6* mutant compared to wild type during conidial development. Phenotypic observation of deletion mutants for other genes with unknown function down-regulated by *ada-6* deletion revealed that deletion mutants for four genes (NCU00929, NCU05260, NCU00116, and NCU04813) produced less conidia than wild type. Deletion of *ada-6* resulted in female sterility, which might be due to that ADA-6 affects oxidation-reduction process and transmembrane transport process, and positively regulates the transcription of *pre-2*, *poi-2*, and NCU05832, three key genes participating in sexual development. In both conidiation and the sexual development process, ADA-6 regulates the transcription of *cat-3* and other genes participating in reactive oxygen species production according to RNA-seq data, indicating a role of ADA-6 in oxidative stress response. This was further confirmed by the results that deletion of *ada-6* led to hypersensitivity to oxidants H_2_O_2_ and menadione. Together, these results proved that ADA-6, as a global regulator, plays a crucial role in conidiation, sexual development, and oxidative stress response of *N. crassa*.

## Introduction

Conidial production is critical for reproduction, dispersal and survival in many filamentous fungi. Sexual reproduction is a key feature that distinguishes eukaryotic organisms from prokaryotic organisms. It produces better-adapted progenies by driving genetic recombination and eliminating deleterious mutations (Ni et al., 2011; Heitman et al., 2013). *Neurospora crassa* is a multicellular ascomycete fungus in the family Sordariomycetes and has long been used as an excellent model organism for genetic and biochemical researches as well as the study of morphological development (Springer, 1993; Perkins and Davis, 2000; Davis and Perkins, 2002). From vegetative growth to conidiation or sexual reproduction, morphological changes were evident. Behind it, transcriptional levels of many genes are altered (Greenwald et al., 2010; Wang et al., 2012; Lehr et al., 2014). For example, 25% predicted genes in the genome of *N. crassa* are differentially expressed during conidiation (Greenwald et al., 2010), as well as during sexual development (Lehr et al., 2014). Transcription factors play important roles in activating or repressing gene expression in response to developmental signals. Thus, identification of transcription factors, which are crucial to conidial and sexual development, and characterization of their mechanisms are critical steps toward deeper understanding of how fungal morphogenesis is regulated.

Several transcription factors required for basal hyphae growth, asexual sporulation, and sexual development have been reported in *N. crassa* (Colot et al., 2006; Carrillo et al., 2017). Among identified 273 transcription factor genes, 33 genes were found specifically affect asexual development (Carrillo et al., 2017). Some of these genes have been extensively studied. For example, *fl* is required for the formation of major constriction chains (Matsuyama et al., 1974; Bailey and Ebbole, 1998). Overexpression of *fl* under the control of a heterologous promoter is sufficient to induce conidiation in a liquid medium which is unfavorable for conidiation (Bailey-Shrode and Ebbole, 2004). Some transcription factor genes, such as *hsf-2*/NCU08480 (Thompson et al., 2008) and *chc-1*/NCU00749 (Sun et al., 2011), were found to regulate the extent of conidiation or conidiation in response to environmental conditions. Deletion of *hsf-2* does not affect hyphal growth and aerial hyphal development but dramatically reduces conidial yield (Thompson et al., 2008). CHC-1 is involved in CO_2_-mediated conidiation suppression, and *chc-1* deletion results in earlier conidial formation than wild type, especially at a higher CO_2_ concentration (Sun et al., 2011). *N*. *crassa* is heterothallic with two mating types, designated *mat a* and *mat A*. Both of the mating types can form protoperithecia when nitrogen source is depleted (Davis and de Serres, 1970). Among identified transcription factor genes, ten of them were found to specifically affect sexual development (Carrillo et al., 2017). For example, *ff-7*/NCU04001 is required for initiation of sexual development, and deletion mutant of *ff-7* does not produce protoperithecia, perithecia as well as ascopores (Colot et al., 2006; Carrillo et al., 2017); Deletion of *bek-1*/NCU00097 results in aberrant perithecia, which exhibit defective beaks and cannot produce ascopores (Colot et al., 2006; Carrillo et al., 2017).

In addition to these specific transcriptional regulators, some transcription factors were showed to participate in regulating both asexual sporulation and sexual development. Knockout mutants of 25 transcription factor genes display significant defects in both asexual sporulation and sexual development (Colot et al., 2006; Carrillo et al., 2017). Most of these genes were named as *all development altered (ada)* genes (Colot et al., 2006), including *ada-1*/NCU00499, *ada-2*/NCU02017, *ada-3*/NCU02896, *ada-4*/NCU03320, *ada-5*/NCU03931, *ada-6*/NCU04866 and *ada-7*/NCU09739. These transcription factors play crucial roles in fungal growth and development by regulating gene expression on a global scale. However, the regulatory roles of most of these newly found transcription factors in growth and development needs further confirmation, and their molecular mechanisms are not addressed. Among them, *ada-6*/NCU04866 is a representative gene, which codes a Zn(II)2Cys6 transcription factor (Borkovich et al., 2004). Deletion of *ada-6* results in slower growth, dramatic reduction in conidiation and female infertility during sexual development (Colot et al., 2006), suggesting ADA-6 is an important transcription factor. However, the confirmation of its function is still required and their mechanism has not been investigated.

ADA-6 orthologs are widely distributed in filamentous fungi by sequence alignment^1^. The ortholog of ADA-6 in *N. discreta* has a predicted function involved in embryonic development^2^. In *Aspergillus oryzae*, the ADA-6 ortholog has predicted role in hyphal growth, positive regulation of secondary metabolite and sporocarp development during sexual reproduction^3^.

In this study, we confirmed the positive role of ADA-6 in growth and development by detailed phenotypic characterization of its deletion mutant and the complemented strain. By comparing transcriptomic profiles generated by RNA-seq from the Δ*ada-6* mutant and wild type during conidiation and sexual development, as well as by analyzing the contribution of the genes or biological pathway influenced by *ada-6* deletion, we explored the mechanisms by which ADA-6 promotes conidiation and sexual development. We also found that deletion of *ada-6* causes hypersensitive to oxidants H_2_O_2_ and menadione. Together, our results proved that ADA-6 is a global regulator of conidiation, sexual development and oxidative stress response in *N. crassa*.

## Materials and Methods

### Strains and Media

Most strains of *N. crassa* used in this study, including FGSC#4200 (wild type), FGSC#11022 (Δ*ada-6*/NCU04866; a), and knockout mutants for genes responsive to *ada-6* deletion, were purchased from the Fungal Genetics Stock Center. All the strains were cultured at 28°C if it’s not mentioned.

Media used in this study include Vogel’s slant medium (1 × Vogel’s salts, 2% sucrose, and 1.5% Bacto Agar), Vogel’s plate medium (1 × Vogel’s salts, 2% glucose, and 0.75% Bacto Agar), liquid Vogel’s medium (1 × Vogel’s salts, 2% glucose), the agar medium for transformant regeneration (1 × Vogel’s salts, 1 M sorbitol, 1 × FGS, and 1.5% Bacto Agar), and the agar medium for filling race tubes [1 × Vogel’s salts, 2% carbon source (glucose, sucrose, xylose, xylan, or carboxymethyl cellulose sodium), and 1.5% Bacto Agar].

### Complementation of the *ada-6* Deletion Mutant

The plasmid pCB1532-ada6 used for complementation was created by inserting a 4547 bp DNA fragment, containing the *ada-6* gene (2230 bp) flanked by a 1238 bp upstream regulatory region and a 1079 bp downstream region, into the plasmid pCB1532 which harbors a sulfonylurea resistant allele of the *Magnaporthe grisea* *ILV*1 as a selective marker (Sweigard et al., 1997). Briefly, the DNA fragment was amplified from the wild-type strain FGSC#4200 using primers Ada6F-EcoRI: GGAATTCGTAAAGTGACTGGAAGGTGG and Ada6R-HindIII: CCCAAGCTTATCAATAACATAACTGCCCCC (*EcoR*I and *Hind*III sites were underlined), digested by *EcoR*I and *Hind*III and ligated into plasmid pCB1532. The construct pCB1532-ada6 was transformed into the Δ*ada-6* mutant FGSC#11022 according to the previously reported protoplast transformation method (Royer and Yamashiro, 1992). 15 μg/ml of chlorimuron ethyl (Sigma) was added to the top agar to inhibit the growth of non-transformed protoplasts. Obtained transformants were subjected to serial transfers on slants with 15 μg/ml chlorimuron ethyl to favor homokaryon formation (Ebbole and Sachs, 1990) and further verified by PCR.

### Analysis of Hyphal Growth, Conidiation, and Sexual Development

Hyphal extension of wild type and the Δ*ada-6* mutant were analyzed in race tube. Briefly, one piece of mycelium mat (2 mm × 10 mm) for each strain was separately inoculated on one end of the race tube containing solid Vogel’s medium with different carbon source [2% glucose, 2% sucrose, 2% xylose, 2% xylan, and 2% carboxymethyl cellulose sodium (CMC-Na), respectively]. Inoculated race tubes were incubated at 28°C and the leading edge of the colony were marked every 24 h. The hyphal extension was then documented and measured by a ruler.

For conidiation analysis, mycelium for each strain was inoculated on Vogel’s slants and grown at 28°C with continuous light for 7 days. Conidia produced were washed by 5 ml distilled water and counted with a hemocytometer.

For protoperithecium and perithecium formation analysis, the mycelial mat or conidia suspension of the strain used as female parent was first inoculated on solid synthetic crossing medium with 0.1% sucrose and grown for 5 days under constant darkness at 25°C. Then the opposite mating-type strain was inoculated as male parent and incubated at 25°C for another 7 days under constant darkness. The protoperithecium and perithecium formation were checked and documented by an optical microscope equipped with a Zeiss CCD.

### Transcriptomic Profiling Analysis

Genome-wide transcriptional profiles for wild type and the Δ*ada-6* mutant during conidiation, and at the initiate stage of protoperithecium formation were obtained by RNA sequencing, while transcriptional profiles for vegetative growth were used as control. Briefly, *N. crassa* wild-type strain and the Δ*ada-6* mutant were inoculated on Vogel’s plates covered with cellophane and grown at 28°C in darkness for 24 h. The mycelia were then transferred into 150-ml flasks containing 75 ml of liquid Vogel’s medium. Cultures were incubated at 28°C with constant agitation at 180 rpm for 18 h, and the mycelia were harvested by vacuum filtration. For conidial development analysis, the mycelial mats were transferred onto the surface of agar plates (9 cm) to induce conidial development at 28°C under constant light. Cultures were sampled at 12 h intervals. For sexual development analysis, the mycelial mats were inoculated on solid synthetic crossing medium with 0.1% sucrose and grown for 4 days under constant darkness at 25°C. Then, the mycelia were harvested and total RNA was extracted according to the standard TRIzol protocol (Invitrogen Corporation, Carlsbad, CA, United States).

RNA samples were sent to Beijing Genomics Institute (BGI) for RNA-seq analysis using the Illumina Hiseq2000 with a 50 bp single-end module (Illumina, San Diego, CA, United States). The obtained raw data was treated, mapped to *N. crassa* genome and transformed into expression value following standard BGI workflow. The gene expression level was calculated by using RPKM (Reads per kb per million reads). The differences in gene expression between samples was compared by comparing RPKM values (Grabherr et al., 2011), and those with fold change more than 2 (FDR < 0.001) were thought to be differentially expressed genes (DEGs). In addition, genes with RPKM less than 12 at all time points were thought to be low abundant transcripts and removed from the DEGs lists. The expressions of some genes, crucial for development and oxidative stress responses, were verified by time course experiment using real time PCR.

### RT-qPCR Analysis

Samples were prepared as described above. Then mycelia were harvested and immediately frozen and ground into fine powder in liquid nitrogen. Total RNA was extracted and treated with DNase I to remove genomic DNA according to the standard TRIzol protocol (Invitrogen Corporation, Carlsbad, CA, United States). cDNA was prepared with a FastQuant RT Kit (with gDNase) (Tiangen, Beijing) according to the product’s instruction. qPCR was performed on a BIO-RAD CFX96^TM^ Real-Time System (Bio-Rad, Hercules, CA, United States) with KAPA SYBR FAST qPCR mix (Kapa Biosystems, Wilmington, MA, United States) according to the product’s protocol. Each cDNA sample was analyzed in duplicate and at least three independent experiments were conducted. The average threshold cycle was used to calculate relative expression level according to 2^-ΔΔCt^ method (Livak and Schmittgen, 2001). And the expression level was normalized to the level of β-tubulin. The primer pairs used for RT-qPCR assay were shown in Supplementary Table S1.

### Susceptibility Tests of the Strains to Oxidative Stress

*N. crassa* wild-type strain and knockout mutants (*ada-6*, *nox-1*, *cat-2*, and *cat-3*) were separately inoculated onto ϕ90-mm plates (containing 15-ml liquid Vogel’s medium) and allowed to grow at 28°C in darkness for 24 h. The mycelial mat were punched and the round mat (ϕ2-mm) were inoculated on the center of plates (ϕ90-mm) with or without oxidant (H_2_O_2_ or menadione), and incubated at 28°C for 22 h (control), 32 h (25 μg/ml menadione) and 48 h (10 mM H_2_O_2_), respectively. Each test was duplicated and the experiment was independently repeated at least three times. The relative growth inhibition rates (mutant growth under oxidant stress was compared to wild type grown under oxidant stress and normalized by growth under non- oxidant condition) of each strain were calculated based on colony diameters after 22 h of incubation.

## Results

### Phenotypic Characterization of the Δ*ada-6* Mutant

**FIGURE 1:**
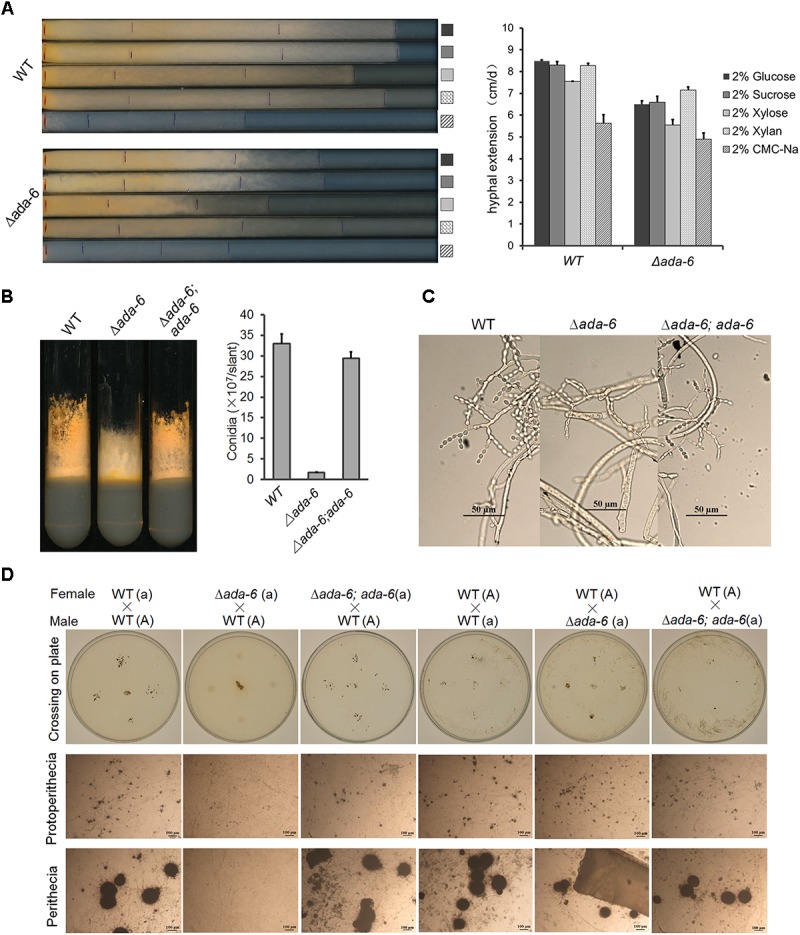
Deletion of *ada-6* results in reduced hyphal growth, less conidial production, and female sterility in *N. crassa*. **(A)** Hyphal growth characterization of the *ada-6* deletion mutant (Δ*ada-6*) and wild type (WT) grown with different carbon source. Strains were grown in race tube containing solid Vogel’s medium with different carbon sources. Inoculated race tubes were incubated at 28°C and the leading edge of the colony were marked every 24 h. The hyphal extension was then measured by a ruler. The means of hyphal extension rates from three race tubes are shown and standard deviations are indicated by error bars. **(B)** Conidiation characterization of wild type (WT), the *ada-6* deletion mutant (Δ*ada-6*) and it’s complemented transformant (Δ*ada-6; ada-6*). Strains were grown in Vogel’s slants at 28°C with continuous light for 7 days and then imaged. Conidia produced on slants were counted with a hemocytometer. The means of conidial counts from three slants are shown and standard deviations are indicated by error bars. **(C)** Conidiophore structure of wild type (WT), the *ada-6* deletion mutant (Δ*ada-6*) and it’s complemented transformant (Δ*ada-6; ada-6*). Bar, 50 μm. **(D)** Protoperithecium and perithecium formation by crossing of the *ada-6* deletion mutant (Δ*ada-6*, a) with wild type (#2225, A). The *ada-6* deletion mutant (Δ*ada-6*, a) or wild type (#2225, A) were used as female parent and first grown on solid crossing medium for 5 days under constant darkness at 25°C, then the opposite mating type strain was inoculated as male parent and incubated at 25°C for another 7 days under constant darkness. Protoperithecium and perithecium formation was checked and imaged.

The phenotype of the Δ*ada-6* mutant has been described by Colot et al. (2006). Deletion of *ada-6* resulted in reduced hyphal growth and altered asexual and sexual development. However, the deletion of *ada-6* only slightly affected colony growth. In race tubes containing solid Vogel’s medium with different carbon sources, the colony growth of the Δ*ada-6* mutant were slightly slower than that of wild type: with 2% glucose, 2% sucrose, 2% xylose, 2% xylan, and 2% carboxymethyl cellulose sodium (CMC-Na) as carbon sources, the growth rate of the Δ*ada-6* mutant was 6.5, 6.6, 5.6, 7.2, and 4.9 cm/day, respectively, while the growth rate of wild type was 8.5, 8.3, 7.5, 8.3, and 5.6 cm/day, respectively (Figure 1A).

The most dramatic effects caused by *ada-6* deletion were the defects in asexual sporulation and sexual development. On slants containing solid Vogel’s medium with 2% sucrose, aerial hyphal growth of the Δ*ada-6* mutant was only slightly shorter than that of wild type (Figure 1B), but the conidial production of the Δ*ada-6* mutant was reduced by 93% as compared with that of wild type (Figure 1B). Unlike the deletion mutant of *fl* in which conidial development stops at the major constriction formation stage (Bailey and Ebbole, 1998), the Δ*ada-6* mutant was capable to pass through all conidial development stages to produce mature conidia (Figure 1C).

To investigate the contribution of ADA-6 to sexual development, we analyzed the formation of protoperithecium and perithecium on solid synthetic crossing medium. When using the Δ*ada-6* (a) as female parent and wild type FGSC#2225 (A) as male parent, only a very few and small protoperithecium formed but no perithecium was observed (Figure 1D). While using wild type 2225 (A) as female parent and the Δ*ada-6* (a) as male parent, normal protoperithecium and perithecium were produced (Figure 1D). These results suggest that deletion of *ada-6* resulted in female sterility.

To confirm the role of ADA-6 in growth and development, we generated a plasmid pCB1532-ada6 which carries the full length of *ada-6* gene with its regulatory regions as described in Materials and Methods. By transforming this plasmid into the Δ*ada-6* mutant FGSC#11022, complemented transformants (Δ*ada-6*; *ada-6*) were obtained and phenotypically compared the growth and development to wild type FGSC#4200 (WT). As expected, the complemented transformants (Δ*ada-6*; *ada-6*) displayed phenotypes resembling wild-type conidiation and sexual development (Figure 1B–D).

### Genome-Wide Transcriptional Responses to *ada-6* Deletion

To understand the regulatory roles of ADA-6 in growth, conidiation and sexual development, transcriptomic profiles generated by RNA-seq from the Δ*ada-6* mutant and wild type during conidial development or sexual development were compared. Among 9403 detected genes, 330, 441, and 1547 genes were found to be transcriptionally changed for more than two folds upon *ada-6* deletion after conidiation induction for 0, 12, and 24 h, respectively (Supplementary Data S1). For sexual development, 1024 genes were found to be transcriptionally changed for more than two folds upon *ada-6* deletion after 4 days of induction of sexual development (Supplementary Data S1).

To functionally understand the DEGs, functional classification and gene set enrichment analysis were conducted. In the vegetative growth stage (or conidiation induction for 0 h), DEGs between the Δ*ada-6* mutant and wild type were mainly enriched in carbohydrate metabolic process (14 up- and 2 down-regulated genes), xylan catabolic process (5 up- and 2 down-regulated genes) and glucose import (6 up- and 2 down-regulated genes) (Supplementary Table S2). After 12 h of conidiation induction, DEGs between the Δ*ada-6* mutant and wild type were mostly enriched in oxidation-reduction process (30 up- and 6 down-regulated genes) and transmembrane transport process (21 up- and 3 down-regulated genes) (Supplementary Table S2). While after 24 h of conidiation induction, DEGs between the Δ*ada-6* mutant and wild type are mostly involved in oxidation-reduction process (107 up- and 111 down-regulated genes) and single-organism metabolic process (188 up- and 195 down-regulated genes) (Supplementary Table S2). During sexual development, DEGs between the Δ*ada-6* mutant and wild type mostly participated in oxidation-reduction process (41 up- and 66 down-regulated genes) and transmembrane transport process (42 up- and 34 down-regulated genes) (Supplementary Table S2).

Under all tested conditions, the expression of 14 genes (*cat-3*, NCU02910, NCU03323, NCU04917, NCU5126, NCU05230, *sut-28*, NCU06170, NCU07088, NCU07095, *cdt-2*, NCU08223, *pho-3*, *adh-9*) were commonly increased and the expression of 13 genes (NCU00496, NCU00719, NCU05629, NCU05762, NCU05859, NCU06140, NCU08455, *eas*, *gao-1*, NCU09210, NCU10610, NCU11340, NCU17271) were commonly reduced in response to *ada-6* deletion (Tables 1–4). Among these genes, seven genes (*sut-28*, *cdt-2*, NCU17271, NCU10610, NCU05762, NCU05230, NCU05126) encode proteins as integral components of plasma membrane, five genes (*cat-3*, *gao-1*, NCU09210, NCU05762, *adh-9*) are involved in oxidation-reduction process, and nine genes (NCU11340, NCU08455, NCU08223, NCU07088, NCU06170, NCU05859, NCU05629, NCU03323, NCU02310) encode proteins with unknown function (Table 1).

**Table 1:** Genes transcriptionally response to *ada-6* deletion under all tested conditions.

Genes	Function annotation	RPKM Δ*ada6*-0 h	RPKM Δ*ada6*-12 h	RPKM Δ*ada6*-24 h	RPKM Δ*ada6*-4 d	RPKM WT-0 h	RPKM WT-12 h	RPKM WT-24 h	RPKM WT-4 d
Down-regulated genes				
NCU04866	*ada-6*	0	0.046	0.0883	0.0408	7.1396	40.4223	19.06	5.1192
NCU00496	hypothetical protein	1.2303	5.8881	3.8363	1.6779	2.8212	17.7147	17.029	5.7047
NCU00719	hypothetical protein, direct target of ADV-1	5.0315	3.9651	0.4172	2.4077	20.5478	9.5147	2.9358	4.8644
NCU05629	hypothetical protein	0.2144	1.9056	1.126	0.3899	4.8593	24.0909	7.9924	3.4413
NCU05762	hypothetical protein with oxidoreductase activity	0	2.7995	0.768	0.6382	0.1248	12.6732	15.5484	48.4745
NCU05859	hypothetical protein	0.4292	3.301	1.0565	14.3203	0.9269	20.0974	10.7257	30.5766
NCU06140	Ribosome biogenesis protein – Nop58p/Nop5	0.4019	4.3793	0.3957	1.7814	2.0093	22.6131	1.4656	4.6589
NCU08455	hypothetical protein	2.7386	48.0429	4.5419	14.67816	8.6476	206.8643	76.1128	83.6734
NCU08457	*eas*, rodlet protein	18.8768	1248.747	270.4869	25.5233	82.9049	10027.32	17750.16	3584.603
NCU09209	*gao-1*, galactose oxidase-1	7.0806	88.2866	34.087	6.212	41.5362	229.2223	364.781	57.7112
NCU09210	dyp-type peroxidase	20.2343	196.3718	52.1181	14.9053	97.0414	549.4004	363.9373	146.3083
NCU10610	hypothetical protein, ADV-1 target gene	2.8556	17.463	3.6971	4.4715	6.3875	38.4268	12.0849	10.8455
NCU11340	hypothetical protein	0	0.4074	0.1956	3.2504	0.1589	0.4009	0.7726	23.0234
NCU17271	hypothetical protein,	0	1.0222	0.3569	0.3707	0.1087	7.2247	6.9613	15.6349
Up-regulated genes				
NCU00355	*cat-3*, catalase-3,	506.5213	574.7889	75.3423	395.3921	237.5218	167.5816	19.7186	62.2595
NCU02910	hypothetical protein,	4.0495	162.2868	8.8953	24.4277	1.8065	18.1556	1.5146	6.7358
NCU03323	hypothetical protein,	54.8567	49.9469	200.5951	170.4939	18.786	17.1281	24.8718	72.6286
NCU04917	hypothetical protein	10.2662	11.3526	0.967	12.4997	2.6423	1.0812	0.2604	0.0796
NCU05126	hypothetical protein,	46.7857	345.4155	39.8113	152.3996	17.3499	166.6553	7.9653	3.9733
NCU05230	hypothetical protein,	5.8748	4.9093	14.3553	58.097	2.0887	1.83	3.7382	28.0041
NCU05897	*sut-28*, sugar transporter-28,	1.0343	426.2465	56.7384	84.4806	0.5171	196.3384	15.1294	36.3927
NCU06170	hypothetical protein,	126.9334	528.2828	59.6491	114.3088	40.5712	86.7585	18.3686	16.1066
NCU07088	hypothetical protein,	20.7894	434.8265	40.0778	49.252	3.2792	46.1012	8.5424	2.611
NCU07095	similar to peptidase s41 family protein	6.1777	17.0835	17.7064	14.0421	0.5295	6.6802	0.3218	2.3609
NCU08114	*cdt-2*, hexose transporter	1.7284	14.7541	11.7635	4598.378	0.5286	4.7714	3.4605	1849.146
NCU08223	hypothetical protein, ADV-1 target gene	1.878	48.883	118.263	23.4981	0.5723	4.7835	9.3052	2.7113
NCU08643	*pho-3*, Acid phosphatase	88.4357	111.5176	8.885	17.0138	25.8364	37.9268	1.9942	1.9201
NCU09798	*adh-9*, aryl-alcohol dehydrogenase,	47.3846	39.8375	8.9522	43.8845	18.4233	11.511	3.1077	4.2254


*(1) WT, wild type; Δ*ada6*, *ada-6* deletion mutant; -0 h, samples of *N. crassa* in vegetative growth stage; -12, samples of *N. crassa* after 12 h of conidiation induction, -24, samples of *N. crassa* after 24 h of conidiation induction; -4 d, samples of *N. crassa* after 4 days of sexual development induction. (2) Locus numbers and function were annotated according to the *N. crassa* genome assembly (http://fungidb.org/fungidb/). (3) RPKM, Reads Per Kilobase of exon model per Million mapped reads.*

### Genes Regulated by ADA-6 Are Involved in Oxidation-Reduction Process

**FIGURE 2:**
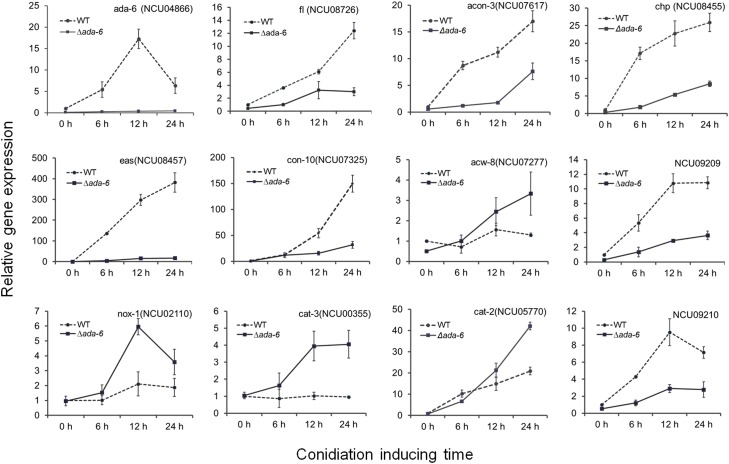
The expressions of some DGEs, crucial for development and oxidative stress responses, were determined by time course experiment using RT-qPCR. Wild type (WT) and the *ada-6* deletion mutant (Δ*ada-6*) were inoculated on Vogel’s plates and allowed to grow at 28°C in darkness for 24 h. The mycelia were then transferred into 150-ml flasks containing 75 ml of liquid Vogel’s medium (2% sucrose). Cultures were incubated at 28°C with constant agitation at 180 rpm for 18 h, and the mycelia were harvested by vacuum filtration and transferred onto the surface of agar plates (9 cm) to induce conidial development at 28°C under constant light. Cultures were sampled after induction for 6, 12, and 24 h. The total RNA was extracted and transcriptional levels of indicated genes were analyzed by RT-qPCR.

During both conidiation and sexual development, the most seriously affected biological process by *ada-6* deletion is oxidation-reduction reaction. After conidiation induction for 12 and 24 h, 36 and 218 genes, respectively, which involved in oxidation-reduction process, were differentially expressed between the Δ*ada-6* mutant and wild type (Supplementary Data S1). The expressions of some crucial genes were verified by time course experiment using real time PCR (Figure 2). During the conidiation induction, the transcriptional levels of 15 genes, involved in oxidation-reduction reaction, were increased in wild type. However, their transcriptional levels in the Δ*ada-6* mutant were obviously lower than those in wild type (Table 2). Most of these 15 DGEs encode oxidase or dehydrogenase, including NCU08856 (myo-inositol oxygenase), NCU05858 (fatty acid oxygenase), NCU09209 (galactose oxidase), NCU10015 (methanesulfonate monooxygenase), NCU04474 (sulfite oxidase), NCU01853 (choline dehydrogenase), NCU03893 (short-chain dehydrogenase/reductase SDR), NCU02287 (acyl-CoA dehydrogenase-1), and three peroxidase encoding genes (NCU09210, *cat-1* and *cat-4*) (Table 2). There were 13 genes in wild type were down-regulated during conidiation, but their transcriptional levers in the Δ*ada-6* mutant were higher than those in wild type. These 13 DGEs include three oxidase encoding genes (NCU06402, NCU04983 and NCU01546), two peroxidase encoding genes (*cat-3* and NCU10051), two dehydrogenase encoding genes (NCU09798 and NCU01754), and two hydrolase encoding genes (NCU01720 and NCU05969), etc. (Table 2).

**Table 2:** Transcriptional responses to *ada-6* deletion by the genes involved in oxidation-reduction process.

Genes	Function annotation	RPKM Δ*ada6*-0 h	RPKM Δ*ada6*-12 h	RPKM Δ*ada6*-24 h	RPKM WT-0 h	RPKM WT-12 h	RPKM WT-24 h
Genes involved in oxidative stress response or with antioxidant activity							
NCU00355	Cat-3	506.5213	574.7889	75.34231	237.5218	167.5816	19.71859
NCU00598	*trx-4*, thioredoxin-4	35.076	62.3528	67.1768	37.2486	67.8260	25.4097
NCU02110	Nox-1	16.33786	44.27099	22.52937	12.94278	22.54903	11.09398
NCU03297	*ccp-1*, cytochrome c peroxidase	179.2891	93.8437	369.5966	195.4672	82.2211	120.0121
NCU03646	L-ascorbate peroxidase	9.7929	48.4457	58.8551	5.0478	47.5253	27.9330
NCU03714	*trx-5*, thioredoxin-5	10.6542	11.0685	90.3788	11.0683	10.8925	16.1468
NCU04268	peroxiredoxin 2 family	301.3897	58.4775	67.2506	274.8588	47.7068	203.4202
NCU05169	Cat-4	6.192728	12.84018	10.87036	5.196066	12.67001	47.45783
NCU05770	Cat-2	42.99698	259.8382	422.92	69.11465	264.3265	149.3483
NCU06556	*trx-6*, thioredoxin-6	424.7498	343.7312	1242.284	344.6572	251.2739	328.9048
NCU06880	*prx-1*, peroxiredoxin-1	145.9389	173.7802	36.7785	152.3986	176.3585	85.6079
NCU07386	*mrp-1*, Fe superoxide dismutase	48.5932	37.0135	15.4648	49.4635	33.9387	33.3602
NCU08791	Cat-1	159.6162	112.2809	200.6002	188.7525	172.519	456.6022
NCU09210	dyp-type peroxidase	20.2343	196.3718	52.1181	97.0414	549.4004	363.9373
NCU09534	*ara-2*, peroxiredoxin HYR1	74.8098	299.9837	261.9622	77.8472	170.2767	87.7308
NCU11046	with predicted peroxidase activity	13.1388	18.1692	128.2936	17.0875	27.9754	56.6314
NCU16942	Peroxidase/oxygenase	0.8903	7.5761	27.7938	0.7507	10.7828	12.9403
Genes involved in other oxidation-reduction process							
NCU01104	ATP-dependent RNA helicase MSS116	5.6171	7.3674	6.1332	6.4801	10.3741	27.1635
NCU01546	coproporphyrinogen III oxidase	972.8109	637.7059	85.5117	958.0831	314.1893	20.422
NCU01720	glycoside hydrolase	161.6695	106.5222	53.2707	151.4619	97.1703	13.9335
NCU01754	*adh-1*, alcohol dehydrogenase-1	4265.2542	2337.2632	287.2989	3179.784	681.5673	175.516
NCU01853	choline dehydrogenase.	0.1569	11.6424	5.4089	0.1255	18.4795	25.5386
NCU02020	Metallo reductase transmembrane component	61.6191	51.6116	18.341	44.9787	23.8857	8.8337
NCU02287	*acd-1*, acyl-CoA dehydrogenase-1	22.8972	179.2092	60.9954	22.7506	293.4145	141.8511
NCU02852	cyp450-31	1.5528	13.122	13.2107	0.8943	15.9223	47.17308
NCU03893	SDR, short-chain dehydrogenase/reductase	99.5548	305.6223	302.4207	138.3449	472.3083	724.727
NCU04474	sulfite oxidase	8.4909	57.1755	8.5554	19.0601	108.0158	76.0423
NCU04865	*pks-3*, polyketide synthase-3	1.48028	1.846	0.4664	1.241	4.3744	9.1439
NCU04924	*cut-1*, phosphatidyl synthase	148.4949	59.6279	580.4246	151.5253	129.0153	1104.3985
NCU04983	lathosterol oxidase	154.7887	126.3888	19.9136	144.1937	54.862	11.0899
NCU05185	NADPH-P450 reductase.	15.7645	22.016	42.4126	12.8737	22.3024	116.3517
NCU05338	hypothetical protein	336.5657	307.6338	354.3336	359.5338	174.4177	17.5311
NCU05858	fatty acid oxygenase.	1.6038	8.8979	6.9641	1.2829	61.5339	24.8003
NCU05969	*gh61-9*, endo-1,4-beta-glucanase	198.02688	156.592	24.2704	76.7552	75.7995	19.4804
NCU06402	C-4 methylsterol oxidase	1578.38	524.4148	183.2211	1578.4012	228.4653	83.66
NCU08856	myo-inositol oxygenase	0.2543	16.4827	9.9163	0.3255	64.2152	25.9664
NCU09209	galactose oxidase	7.0806	88.2866	34.087	41.5362	229.2223	364.781
NCU09798	aryl-alcohol dehydrogenase	47.3846	39.8375	8.9522	18.4233	11.511	3.1077
NCU10051	flavohemoglobin	3568.8831	507.7287	867.6508	3361.9474	186.2688	479.5577


*(1) WT, wild type; Δ*ada6*, *ada-6* deletion mutant; -0 h, samples of *N. crassa* in vegetative growth stage; -12, samples of *N. crassa* after 12 h of conidiation induction; -24, samples of *N. crassa* after 24 h of conidiation induction. (2) Locus numbers and function were annotated according to the *N. crassa* genome assembly (http://fungidb.org/fungidb/). (3) RPKM, Reads Per Kilobase of exon model per Million mapped reads.*

Oxidation-reduction reaction is involved in many processes. The foremost is oxidative stress response pathway, which affect fungal growth, development and stress responses. During the conidiation induction, 15 genes involved in oxidative stress response or with antioxidant activity were differentially expressed between the Δ*ada-6* mutant and wild type (Table 2). After 24 h of conidiation induction, the transcriptional levels of 11 genes (*nox-1*, *cat-2*, *cat-3*, NCU03646, NCU09534, NCU16942, *ara-2*, NCU16942, *ccp-1*, NCU11046, *trx-4*, *trx-5* and *trx-6*) in the Δ*ada-6* mutant were obviously higher than those in wild type. Among these 11 genes, the transcriptional levels of *nox-1* and *cat-3* in the Δ*ada-6* mutant were higher than those in wild type by both 12 and 24 h after conidiation induction (Table 2 and Figure 2). NOX-1 participates in reactive oxygen species (ROS) production, and CAT-3 activity increases during exponential growth and is induced under various stress conditions (Chary and Natvig, 1989; Peraza and Hansberg, 2002). The gene *cat-2* encodes catalase-2, which is mainly found in aerial hyphae and conidia (Peraza and Hansberg, 2002). The coding products of NCU03646, NCU09534, NCU16942, *ara-2*, NCU16942, *ccp-1*, NCU11046 have peroxidase activity, and *trx-4*, *trx-5* and *trx-6* encode thioredoxin. All these genes are induced by ROS or other stresses (Circu and Aw, 2010; Matsuo and Yodoi, 2013). The higher transcription of these 11 genes in the Δ*ada-6* mutant than those in wild type may suggest that the Δ*ada-6* mutant produce more ROS than wild type during the conidiation induction. For the genes *cat-1*, *cat-4*, NCU07386 (*mrp-1*, Fe superoxide dismutase), NCU09210 (dyp-type peroxidase), NCU04268 (peroxiredoxin 2), and NCU06880 (*prx-1*, peroxiredoxin-1), their transcriptional levels in the Δ*ada-6* mutant were obviously lower than those in wild type after 24 h of conidiation induction (Table 2). The transcriptional levels of *cat-1* and *cat-4* were increased in wild type but not changed in the Δ*ada-6* mutant during the late period of conidiation (Table 2). CAT-1 is highly abundant in conidia and function mainly in condia germination (Peraza and Hansberg, 2002; Wang et al., 2007), CAT-4 is located in the cytosol (Schliebs et al., 2006), and both CAT-4 and dyp-type peroxidase (NCU09210) were induced during the conidiation (Greenwald et al., 2010). As expression of these genes is correlated with conidiation, the lower transcription of the *cat-1*, *cat-4*, and NCU09210 in the Δ*ada-6* mutant compared with those in wild type is consistent with the less sporulation phenotype of the Δ*ada-6* mutant.

During sexual development, 107 genes involved in oxidation-reduction process were found differentially expressed between the Δ*ada-6* mutant and wild type (Supplementary Table S2 and Table 4). After 4 days of sexual development induction, the transcriptional levels of 61 genes in wild type were increased or not changed, but were obviously lower in the Δ*ada-6* mutant than in wild type. While the transcriptional levels of 26 genes were obviously higher in the Δ*ada-6* mutant than in wild type. Among these 107 genes, 15 genes are involved in oxidative stress response or with antioxidant activity (Table 4). After 4 days of sexual development induction, the transcriptional levels of 7 genes (*nox-1*, *nox-2*, *nox-R*, *cat-3*, NCU03151, NCU08114, and NCU07966) in the Δ*ada-6* mutant were obviously higher than those in wild type. NOX-1, NOX-2, and NOX-R participate in ROS production, and CAT-3 activity increases during exponential growth and is induced under various stress conditions (Chary and Natvig, 1989; Peraza and Hansberg, 2002). NCU03151 encodes a peroxisomal membrane protein, and NCU08114 (*cdt-2*) encodes a hexose transporter. The higher expression of these seven genes in the Δ*ada-6* mutant suggests that the Δ*ada-6* mutant may be still metabolically active and produced more ROS than wild type after 4 days of sexual development induction. For the genes *sod-1*, *cat-1*, *cat-4*, NCU09210, NCU07966, NCU05858, NCU11286, and NCU03651, their transcriptional levels in the Δ*ada-6* mutant were obviously lower than those in wild type (Table 4). As expression of these genes is correlated with conidiation, the lower expression of these eight genes in the Δ*ada-6* mutant than in wild type is consistent with their phenotypic characteristics, as asexual development is companied with sexual development after 4 days of sexual development induction.

### Genes Regulated by ADA-6 Are Involved in Conidiation and Vegetative Cell Wall Development

Many genes are associated with conidiation in *N. crassa*. Among the genes positively regulating conidiation, *acon-3* and *fl* were differentially expressed between the Δ*ada-6* mutant and wild type (Table 3). The result was verified by time course experiment, in which the expression profiles of *acon-3* and *fl* were analyzed by real time PCR (Figure 2). During the conidiation induction, the transcriptional level of *acon-3* and *fl* in wild type were significantly increased after conidiation induction. At 12 h, the *acon-3* and *fl* transcriptional level was 11.2 times and 6.1 times higher than that at the initial time point, respectively. However, the transcriptional increases of *acon-3* and *fl* were only 1.7-fold and 3-fold in the Δ*ada-6* mutant after 12 h induction. Similar results were found at 24 h of conidiation induction. The transcriptional levels of *acon-3* and *fl* in the Δ*ada-6* mutant were lower than those in wild type during the entire experiment (Figure 2).

**Table 3:** Transcriptional response to *ada-6* deletion by the genes involved in conidiation and vegetative cell wall development.

Genes	Function annotation	RPKM Δ*ada6*-0 h	RPKM Δ*ada6*-12 h	RPKM Δ*ada6*-24 h	RPKM WT-0 h	RPKM WT-12 h	RPKM WT-24 h
Genes positively regulating conidiation							
NCU04866	Ada-6	0	0.045997	0.088329	7.139561	40.42227	19.05998
NCU00116	Aab-1, TF subunit	41.0876	104.1344	177.4739	48.6871	114.6333	560.9975
NCU00269	Set-2	1.868055	2.594838	22.58605	2.667073	3.150217	6.208719
NCU00929	hypothetical protein	9.3544	4.7866	7.945482	7.22975	4.6107335	25.61734
NCU04813	hypothetical protein	13.3412	12.7783	9.8237	13.9730	15.9312	27.8958
NCU05260	Protein kinase	7.46533	3.77964	5.44357	6.425309	4.244103	19.1833
NCU07617	Acon-3	0.186927	1.389872	3.91145	0.635499	8.159488	4.862642
NCU08726	*fl*, fluffy	9.121856	17.91114	14.32059	17.76177	36.69432	45.58477
Genes which deletion mutants with enhanced conidial production							
NCU00355	Cat-3	506.5213	574.7889	75.34231	237.5218	167.5816	19.71859
Genes preferentially expressed during conidiation							
NCU07324	Con-13	0.300536	2.568501	2.56481	0.480817	7.380787	57.28341
NCU07325	Con-10	10.91534	72.60819	263.9326	7.64009	77.11508	984.083
NCU08457	Eas	18.87683	270.4869	1248.747	82.90488	10027.32	17750.16
NCU08769	Con-6	3.702609	38.78643	69.66419	1.057798	72.8696	2500.538
NCU09235	Con-8	12.17338	203.7125	271.1714	26.04317	922.9603	1041.627
NCU09357	stage V sporulation protein K	0.651945	13.99727	7.899063	3.809303	11.22863	23.29247
Genes encoding vegetative cell wall protein							
NCU05974	Mwg1	224.3592	118.3476	191.3125	193.3397	63.22570	86.4393
NCU07277	Acw-8	569.5978	480.884	407.5197	517.1306	321.3095	64.3942


*(1) WT, wild type; Δ*ada6*, *ada-6* deletion mutant; -0 h, samples of *N. crassa* in vegetative growth stage; -12, samples of *N. crassa* after 12 h of conidiation induction; -24, samples of *N. crassa* after 24 h of conidiation induction. (2) Locus numbers and function were annotated according to the *N. crassa* genome assembly (http://fungidb.org/fungidb/). (3) RPKM, Reads Per Kilobase of exon model per Million mapped reads.*

**FIGURE 3:**
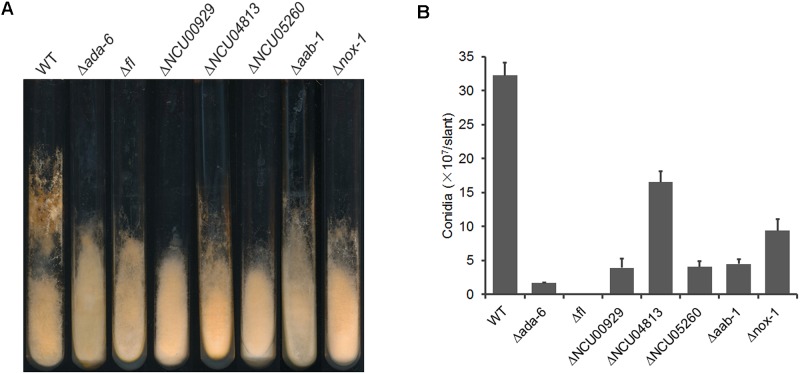
Conidial production of the knockout mutants of genes regulated by ADA-6. **(A)** Wild type and knockout mutants were inoculated and grown on Vogel’s slants at 28°C with constant darkness for 1 day, and then transferred to constant light for another 6 days. Conidial production of each strain was documented as images. **(B)** Conidial production was measured as number of conidia per slant. Standard deviations from three replicates were marked by error bars.

For DEGs with unknown functions, we analyzed conidiation of the corresponding gene deletion mutants grown on Vogel’s slants and found that mutants for four genes, including NCU00929, NCU05260, NCU00116, and NCU04813, displayed reduced conidial production (Figure 3). NCU05260 encodes a protein kinase, NCU00116 encodes a CCAAT-binding transcription factor subunit AAB-1 (Chen et al., 1998), and both NCU00929 and NCU04813 encode hypothetical protein. The transcriptional levels of these four genes were increased dramatically at 24 h after conidiation induction, but their transcriptional levels were lower in the Δ*ada-6* mutant than those in wild type (Table 3).

Some conidiation related genes, including *eas*, *con-6*, *con-8*, *con-10*, *con-13*, and NCU09357 (encoding stage V sporulation protein K), were highly expressed in wild type during the late period of conidiation (Table 3 and Figure 2). However, the transcriptional levels of these genes in the Δ*ada-6* mutant were much lower than those in wild type in the mid-late period of conidiation (Table 3 and Figure 2). This result is consistent with the phenotype of reduced conidial production in the Δ*ada-6* mutant. After the 24 h of conidiation induction, the wild type had produced many spores, while the Δ*ada-6* mutant produced only a few spores (data not shown). In consistent with this phenotype, the genes encoding cell wall proteins in vegetative hyphae, including *acw-8* and *mwg1* (Maddi et al., 2009), were higher expressed in the Δ*ada-6* mutant than those in wild type in the mid-late period of conidiation (Table 3 and Figure 2).

Some genes negatively influence conidial development and their deletion resulted in earlier or enhanced conidial production (Michán et al., 2003; Sun et al., 2011, 2012). Among these genes, *cat-3* was differentially expressed between the Δ*ada-6* mutant and wild type (Table 3 and Figure 2). As shown in Figure 2, the expression of *cat-3* was not increased during conidiation in wild type. However, the transcriptional level of *cat-3* in the Δ*ada-6* mutant was higher than those in wild type during the entire period of conidiation induction.

### Regulation of the Genes Involved in Sexual Development by ADA-6

A large number of *N. crassa* genes have been identified to be required for sexual development. Among them, only five genes (*app*, *poi-2*, *pre-2*, *fbm-1*, and NCU05832) were found differentially expressed between the Δ*ada-6* mutant and wild type during sexual development according to RNA-seq data (Table 4). NCU05832 encodes a methyltransferase, whose homologue in *A. fumigatus* negatively regulates sexual sporulation, and its deletion resulted in formation of a cellular spore^4^. The transcription of NCU05832 was not induced in wild type, but induced in the Δ*ada-6* mutant during sexual development. After 4 days of sexual development induction, the transcriptional level of NCU05832 in the Δ*ada-6* mutant was 142% higher than that of wild type (Table 4). This result indicates that NCU05832 is negatively regulated by ADA-6.

**Table 4:** Transcriptional response to *ada-6* deletion by the genes involved in sexual development and oxidative stress response.

Locus	Function annotation	RPKM Δ*ada6*-0 h	RPKM Δ*ada6*-4 d	RPKM WT-0 h	RPKM WT-4 d
Genes participating in sexual development							
NCU00552	*al-1*, phytoene desaturase	31.6069	2478.7951	51.6463	990.8349
NCU01427	*al-3*, geranylgeranyl pyrophosphate synthetase	11.8371	605.0731	16.8135	120.2416
NCU02925	*fbm-1*, fruiting body maturation-1	0.9891	23.4505	0.0609	836.3750
NCU04533	App	5.3825	8.0260	4.7362	1253.4752
NCU05758	*pre-2*, pheromone receptor	0.1754	12.2255	0.3274	31.7476
NCU05768	*poi-2*, mating response protein POI2	4.0454	8.0569	1.0016	2263.0728
NCU05832	methyltransferase	81.2262	225.2263	89.1113	93.2263
Genes involved in oxidative stress response							
NCU00355	*cat-3*, catalase-3	506.5213	395.3921	237.5218	62.2595
NCU02110	*nox-1*, NADPH oxidase 1	16.3379	40.627793	12.9428	13.0752
NCU02133	*sod-1*, superoxide dismutase	491.3552	58.7633	550.0388	220.3283
NCU03151	Peroxisomal membrane protein	304.0955	210.8447	326.5764	99.0474
NCU03651	*mdh-2*, malate dehydrogenase-2	121.4947	13.5248	117.2605	40.4284
NCU05169	*cat-4*, catalase-4	6.1927	7.6502	5.1961	19.9277
NCU05858	*fam-2*, fatty acid oxygenase	1.6038	8.1275	1.2828	39.4565
NCU07850	*nor-1*, NoxR	68.7881	32.1234	61.9067	15.5760
NCU07966	*trm-1*, calcium-transporting ATPase 3	0.9297	183.4750	0.9405	75.4148
NCU08114	*cdt-2*, hexose transporter	1.7284	4598.3782	0.5286	1849.1457
NCU08791	*cat-1*, catalase-1	159.6162	33.7654	188.7525	192.4185
NCU09210	dyp-type peroxidase	20.2343	14.9053	97.0414	146.3083
NCU10775	*nox-2*, NADPH oxidase 1	94.5026	30.2639	81.3519	12.8031
NCU11286	Peroxidase/oxygenase	0.0001	0.1906	0.0001	20.0431


*(1) WT, wild type; Δ*ada6*, *ada-6* deletion mutant; -0 h, samples of *N. crassa* in vegetative growth stage; -4 d, samples of *N. crassa* after 4 days of sexual development induction. (2) Locus numbers and function were annotated according to the *N. crassa* genome assembly (http://fungidb.org/fungidb/). (3) RPKM, Reads Per Kilobase of exon model per Million mapped reads.*

The *app* (*a*bundant *p*erithecial *p*rotein) is an indicator of sexual development, and its transcripts occur only after the onset of sexual development (Nowrousian et al., 2007). *pre-2* (NCU005758) is a pheromone receptor encoding gene, and plays a vital role during mating in *N. crassa* (Kim and Borkovich, 2006). *poi-2* is essential for differentiation of female reproduction structures and perithecial development (Kim and Nelson, 2005). *fbm-1* encodes fruiting body maturation-1, whose homologue in *N. discreta* is Kynurenine 3-monooxygenase and related to flavoprotein monooxygenases^5^. The transcripts of *app*, *poi-2*, *pre-2*, and *fbm-1* increased during sexual development, but were obviously lower in the Δ*ada-6* mutant than those in wild type. After 4 days of sexual development induction, transcriptional level of *app*, *poi-2*, *pre-2*, and *fbm-1* in the Δ*ada-6* was only 0.6, 0.4, 38.5, and 2.8%, respectively, of that in wild type (Table 4). This result suggests that ADA-6 positively regulates the transcription of *pre-2* and *poi-2*, which promote sexual development. The lower expression of *app* and *fbm-1* in the Δ*ada-6* mutant might be the consequence of female sterility of the Δ*ada-6* mutant.

### Deletion of *ada-6* Causes Hypersensitivity to Oxidants

**FIGURE 4:**
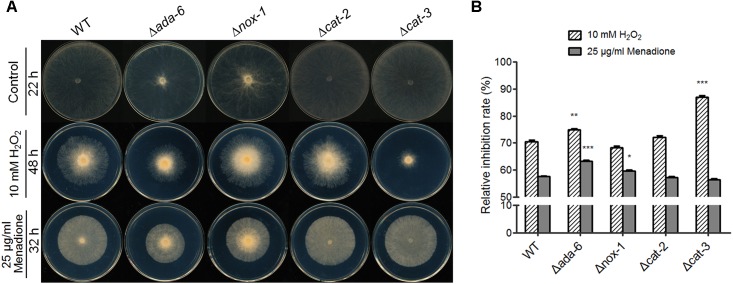
Susceptibility test of *N. crassa* wild type and knockout mutants (*ada-6*, *nox-1*, *cat-2*, and *cat-3*) to oxidants. **(A)** Susceptibility test of the strains to oxidants. Wild type and the mutants were inoculated in ϕ90-mm plates (containing 15-ml liquid Vogel’s medium) and allowed to grow at 28°C in darkness for 24 h. The mycelial mat were punched and the round mat with ϕ4-mm were inoculated in the center of plates (ϕ-90 mm) with or without oxidant (H_2_O_2_ or menadione), and incubated at 28°C for indicated time, respectively. Each test was duplicated and the experiment was independently repeated at least three times. **(B)** Relative growth inhibition rates were calculated based on colony diameters after 22 h of incubation. Values from three replicates were used for a statistical analysis. Means of the inhibition rates are shown, and standard deviations are marked with error bars. Differences between the mutants and the WT were statistically analyzed by the analysis of variance. Values with *P* < 0.001, 0.001 < *P* < 0.01, and 0.01 < *P* < 0.05 are marked with ^∗∗∗^, ^∗∗^, and ^∗^, respectively.

During both conidiation and sexual development, RNA-seq data suggest that ADA-6 regulate the transcription of *cat-3* and genes participating in ROS production. If it is true, deletion of *ada-6* might cause an alteration in the sensitivity to oxidants. To confirm this, we inoculated knockout mutants (*ada-6*, *nox-1*, *cat-2*, and *cat-3*) and wild type on plates with or without oxidants (10 mM H_2_O_2_ or 25 μg/ml menadione), and the relative growth inhibition rates of each strain were calculated after 22 h of incubation. The Δ*cat-3* strain displayed hypersensitivity phenotype to H_2_O_2_ (Figure 4A): the relative growth inhibition of the Δ*cat-3* strain (87%) is higher than that of wild type (70%) (Figure 4B), while, the sensitivity of the Δ*cat-3* mutant to menadione was similar to that of wild type (Figure 4). This result is consistent with previous report (Michán et al., 2003). The sensitivity of the Δ*cat-2* mutant to both H_2_O_2_ and menadione was similar to wild type, and the *Δnox-1* mutant showed slight hypersensitive to menadione and similar sensitive to H_2_O_2_ with wild type (Figure 4). The Δ*ada-6* mutant was more sensitive than wild type to both H_2_O_2_ and menadione (Figure 4A). On the plates with 10 mM H_2_O_2_, the growth of the Δ*ada-6* mutant and wild type was inhibited by 75 and 70%, respectively. On the plates with 25 μg/ml menadione, the growth of the Δ*ada-6* mutant and wild type was inhibited by 63 and 58%, respectively. Above results, together with RNA-seq data suggest that ADA-6 might play a role in oxidative stress response.

## Discussion

Global transcription factors control fungal growth, development and stress responses by regulating gene transcription on a global scale. Several transcription factors encoding genes named as *all development altered (ada)* were previously identified, and their deletion resulted in significant defects in basal hyphal growth, asexual sporulation, and sexual development (Colot et al., 2006; Carrillo et al., 2017). One of them is *ada-6* (NCU04866), whose deletion resulted in slower growth, dramatic reduction in conidiation and female infertility (Colot et al., 2006). However, the regulatory role of ADA-6 in growth and development needs further confirmation by mutant complementation, and its mechanism has not been addressed. Here we first confirmed the positive role of ADA-6 in growth and development by detailed phenotypic characterization of its deletion mutant and the complemented mutant. Then, by comparing transcriptomic profiles and functional analysis of genes influenced by *ada-6* deletion, we explored the mechanisms by which ADA-6 promotes conidiation and sexual development. Our results demonstrate that ADA-6 might play a role in conidiation by regulating oxidation-reduction process and single-organism metabolic process. Moreover, ADA-6 positively regulates the transcription of *fluffy* and *acon-3*, two key genes required for the initiation of asexual sporulation by controlling the formation of major constriction chains (Matsuyama et al., 1974; Springer and Yanofsky, 1989; Bailey and Ebbole, 1998; Bailey-Shrode and Ebbole, 2004; Chung et al., 2011). Deletion of *ada-6* also resulted in female sterility. ADA-6 regulates some genes associated with sexual development, including *pre-2*, *poi-2*, and NCU05832, three key genes required for sexual development (Kim and Nelson, 2005; Kim and Borkovich, 2006).

Oxidation-reduction reaction is involved in many pathways. The foremost of these is oxidative stress response pathway, which affects fungal growth, development and stress responses. ADA-6 regulates the transcription of *cat-3* and genes participating in ROS production according to RNA-seq data, indicating a role of ADA-6 in oxidative stress response. This was confirmed by the hypersensitivity phenotype of the Δ*ada-6* mutant to oxidants H_2_O_2_ and menadione.

Numerous studies have found the role of ROS in the regulation of conidiation and sexual development (Michán et al., 2003; Aguirre et al., 2005; Cano-Domínguez et al., 2008). In *N. crassa*, formation of conidia from growing hyphae includes three morphogenetic developmental stages: growing hyphae to adherent mycelium, adherent mycelium to aerial hyphae, and aerial hyphae to conidia. A hyperoxidant state develops at the start of these morphogenetic transitions (Hansberg et al., 1993; Toledo et al., 1995). Oxidative stress due to lack of CAT-3 induces hyphal adhesion, and development of more aerial hyphae and conidia (Michán et al., 2003). NOX-1, NOX-2, and NOX-R participates in ROS production, and NOX-1 elimination results in complete female sterility, decreased asexual development, and reduction of hyphal growth in *N. crassa* (Cano-Domínguez et al., 2008). All these studies indicate that ROS, whose accumulation is induced by eliminating ROS-decomposion (CAT-3) or activating ROS-generation (NOX-1), is a critical cell differentiation signal promoting conidiation and sexual development. In our study, ADA-6 regulates the transcription of oxidative stress related genes, including *nox-1*, *cat-3*, etc., during both conidiation and sexual development. Based on above result, we speculate that more ROS may accumulate in cells of the Δ*ada-6* mutant during development or stress response process. This was further confirmed by the results that deletion of *ada-6* resulted in hypersensitive to oxidative stress inducers H_2_O_2_ and menadione. Based on previous studies, this ROS accumulation can promote conidiation and sexual development in *N. crassa* (Hansberg et al., 1993; Toledo et al., 1995; Cano-Domínguez et al., 2008). However, the Δ*ada-6* mutant still exhibited reduced conidial production and female sterility. These results indicate that regulation of development and oxidative stress response by ADA-6 may be independent.

In summary, our study showed that ADA-6, as a global regulator, plays positive roles in conidiation and sexual development, and regulates oxidative stress response of *N. crassa*. Combining transcriptomic profiles and functional assay, we explored the mechanisms by which ADA-6 promotes conidiation and sexual development, and regulates oxidative stress response. This work has augmented our knowledge of the functions and mechanisms of global regulators that influence growth, development and stress responses in filamentous fungi.

## Author Contributions

XS designed the study and wrote the manuscript. XS, FW, NL, and CH performed the main experiments. SL, BL, WX, and ZZ contributed to the data analysis and the data interpretation.

## Conflict of Interest Statement

The authors declare that the research was conducted in the absence of any commercial or financial relationships that could be construed as a potential conflict of interest.

## Footnotes

1 http://fungidb.org/fungidb/app/record/gene/NCU04866#evolutionary-biology

2 http://fungidb.org/fungidb/app/record/gene/NEUDI_104878

3 http://fungidb.org/fungidb/app/record/gene/AO090010000113#GOTerms

4 http://fungidb.org/fungidb/app/record/gene/NCU05832#evolutionary-biology

5 http://fungidb.org/fungidb/app/record/gene/NCU02925#evolutionary-biology
