# Supplementary material for: The Zn(II)2Cys6-Type Transcription Factor ADA-6 Regulates Conidiation, Sexual Development, and Oxidative Stress Response in Neurospora crassa
Source: Front Microbiol. 2019 Apr 10;10:750. doi: 10.3389/fmicb.2019.00750 (PMC6468284; doi:10.3389/fmicb.2019.00750)
Supplement: Supplementary file 2 [file Table_2.DOCX]

**Supplementary Table S1**

**Table S1.** Gene-specific primer pairs used for quantitative real-time RT-PCR assay

| Gene | | Primers sequence (5’→3’) | | Gene | | Primers sequence (5’→3’) | |
| --- | --- | --- | --- | --- | --- | --- | --- |
| NCU08455  (*chp*) | QchpF: CTTCAACACCAAATTCCTCTTCAG  QchpR: CTTCACCCATAATTCGCACAC | | NCU00355  (*cat-3*) | | Qcat3F: TTATTGGCACTGCTCTAGCGGCAT  Qcat3R: TGTCGTCAACCTCAACCTCCTTCA | |  |
| NCU08726  (*fl*) | QflF: AGACTACGAAACGATGCCAACCCT  QflR: TAGAGAACGGCCGCAAAGAGCATA | | NCU05770  (*cat-2*) | | Qcat2F: TACAAGTTTGACTGGGAGCCGACA  Qcat2R: ACATGGTCGGGAGCTTCTTCTTGT | |  |
| NCU07617  (*acon-3*) | Qacon3F: GAGTTTGTCGAGCATCAGC  Qacon3R: GGTATCCGTATCCATTGCCG | | NCU09209  (*gao-1*) | | QN9209F: ACCATACCCTCTCAATCCTG  QN9209R: CCGTTCTCTGTCAGGAGATAC | |  |
| NCU08457  (*eas*) | QeasF: ACAAGCCTTACTGCTGCCAGTCTA  QeasR: AACATCGTCCTTGCAGCACTTGAC | | NCU09210 | | QN9210F: GTCGAACTCGTCCTGAAAAG  QN9210R: CCTTTGGCTCTTTGTCCTTG | |  |
| NCU07325  (*con-10*) | Qcon10F: CCAAGGAAGAGGTTCAGGCCAT  Qcon10R: TTGCCGCCCTTGGAAGCAATTT | | NCU04866  (*ada-6*) | | Qada6F: TTCAAGGCACACATCTTCCTCCCT  Qada6R: TGGATTTCGCCGTCGTATTCGGTT | |  |
| NCU07277  (*acw-8*) | QN7277F: ACTACCACTCACACTATCACC  QN7277R: AGGAAGTAGGCTCAACAGG | | *β-tub* | | QbtubF: CCCAAGAACATGATGGCTGCTTCT QbtubR: TTGTTCTGAACGTTGCGCATCTGG | |  |
| NCU02110  (*nox-1*) | Qnox1F: CGTATTTGAATGGGTTGGAGG  Qnox1R: CCTAAAATTGACCTCACGAGTC | |  | |  | |  |
